# Chronic disseminated intravascular coagulation presenting as renal mass

**DOI:** 10.4103/0971-9261.44767

**Published:** 2008

**Authors:** Manjiri Somashekhar, Padmalatha S. Kadamba, Mugdha Wakodkar

**Affiliations:** Department of Paediatric Surgery, MS Ramaiah Medical and Teaching Hospital, Bangalore, India

**Keywords:** Chronic disseminated intravascular coagulation, Wilm's tumor, renal mass

## Abstract

Disseminated intravascular coagulation (DIC) is a complex clinical syndrome, described as a sequential activation of the coagulation and fibrinolytic system. Trauma and sepsis are some of the known precipitating factors. We report a case of nonovert disseminated intravascular coagulation presenting as a huge renal mass in a 3-year-old child, suspected to be a Wilms’ tumor. On imaging studies, it was found to be a renal hematoma. Laboratory investigations revealed an underlying chronic disseminated intravascular coagulation caused by sepsis. The child recovered with conservative treatment; follow up investigations showed resolution of renal hematoma with renal function returning to base line. Clinical presentation of Chronic DIC is variable. Laboratory investigations usually help to diagnose the condition and also to monitor the progress of the treatment. The treatment of the triggering cause is the cornerstone of the management of this condition.

## INTRODUCTION

Chronic disseminated intravascular coagulation (DIC), also known as compensated disseminated intravascular coagulation, results from a persistent weak or intermittent activating stimulus. Under such conditions, destruction and production of coagulation factors and platelets are balanced.[1] Chronic DIC is usually associated with carcinomatosis, retained dead fetus, liver disease, aneurysm or hemangioma.[2] Sepsis usually causes acute disseminated intravascular coagulation but nonovert chronic DIC is also observed.[1] Liver hematoma, subdural hematoma[3,4] and post-biopsy renal hematoma due to chronic disseminated intravascular coagulation have been reported.[5] We are reporting an unusual presentation of chronic DIC in the form of a renal mass.

## CASE REPORT

**Figure 1:**
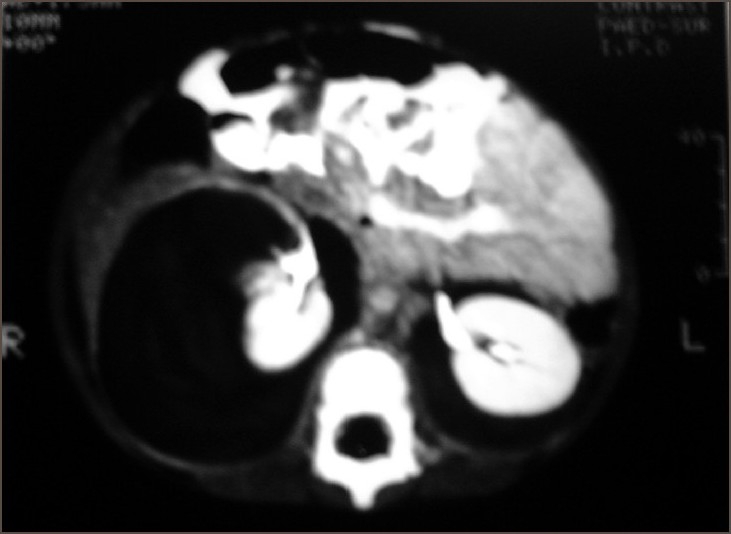
CT scan showing right renal hematoma

A 3-year-old male child was admitted with complaints of high-grade continuous fever in addition to painful swelling of right leg, jaundice and abdominal distention, gradually progressing over 20 days. Prior to this, there was a history of trivial trauma over the abdomen. In addition, a wound discharging pus was noticed since 8 days over his right leg. On examination, the child was febrile, tachypneic, pale, icteric and edematous. Abdomen was distended with visible dilated veins and everted umbilicus. A huge 15 × 10 cm firm, tender and fixed mass was palpable occupying the whole of right side of the abdomen. It was suspected to be a renal mass, probably Wilms’ tumor. Auscultation of chest revealed bilateral basal fine crepitations, tachycardia and systolic murmur. His right leg was swollen in its entirety and a distinct 2 × 1 cm tender, sinus discharging pus was seen over the distal one third of his leg. Hematuria was noticed on catheterization. His laboratory investigations revealed the following: hemoglobin of 2 gm%, packed cells volume 28.3 mg/cc, total count of 16,000 cells/cc, platelet 1.82 lacs/cmm, microcytic hypochromic anemia in peripheral smear as well as neutrophilic leukocytosis 80%, and band form cells with retic count 3%. Prothrombin time was 18.8 s (control 16.2 s) and activated partial thromboplastin time 48 s (control 26.5 s), fibrin degradation products (FDP) were > 2000 ng/ml. Liver function tests revealed total bilirubin of 2.0 mg/dl, direct bilirubin 1.80 mg/dl, AST 79 mg/dl, ALT 44 mg/dl, alkaline phosphatase 247 mg/dl, and serum albumin was 1.9gms/dl. Renal function was slightly deranged with serum creatinine of 1.2 mg/dl. Serum electrolyte levels were within normal limits: Na 127 meq/L, K 4.9 meq/L and Cl 104 meq/L. Ultrasound [Figure 1] of the abdomen demonstrated a heterogeneous, lamellated, avascular mixed echogenic mass in the right perinephric region measuring 13 × 6 × 6.5 cm with urinary bladder having some echogenic material, probably blood clots. Computerized tomography scan Figure 1 showed evidence of contusion of the right kidney with perinephric hematoma which was confined to renal capsule displacing the ureter anteriorly X-ray of the right leg exhibited changes of osteomyelitis in distal one-third of the tibia. Pus from the wound was sterile. Biopsy of the bone confirmed chronic non-specific osteomyelitis. The patient was managed conservatively with broad spectrum intravenous antibiotics for three weeks. Two units of packed red cells, three units of fresh frozen plasma and two units of cryoprecipitate were transfused over a period of 10 days. Sinus over the right leg was explored, sequestrectomy was done and the limb was immobilized. Follow up investigations showed improvement in hemoglobin and coagulation parameters. He was symptomatically better, with a gradual decrease in abdominal distention and tenderness. A small palpable mass was present in the right lumbar region. The urine output was adequate without hematuria. The child was taking orally and was ambulant with cast in situ. He was discharged at the end of 4 weeks and followed up after 2 weeks. He looked healthy, with a very small nontender mass palpable in the right lumbar region. Follow up ultrasound still demonstrated a 8 × 3 × 5 cm right perinephric hematoma. Intravenous urography revealed normally functioning kidneys. Thrombus was seen in the inferior vena cava between the liver and common iliac veins with multiple retroperitoneal collaterals. He further received 6 weeks treatment with aspirin and oral antibiotics for thrombus and osteomyelitis, respectively. Final diagnosis was of chronic DIC triggered by sepsis due to trauma and chronic osteomyelitis.

## DISCUSSION

DIC is a complex syndrome in which there is pathological generation of thrombin and diffuse intravascular clot formation.[6] It is known to occur as acute decompensated or chronic compensated form. The underlying disease that sparks off DIC determines the clinical presentation. In acute decompensated DIC, there is a sudden massive exposure of tissue factor over a brief time period; it is beyond the capacity of control mechanisms and there is no time for generation of coagulation factors.[7] Chronic DIC, also known as compensated DIC, results from a persistent weak or intermittent activating stimulus. Under such conditions, destruction and production of coagulation factors and platelets are balanced.[1]

Bleeding is a universal manifestation of DIC, but most of the morbidity and mortality of DIC is due to microvascular thrombosis.[7] Thrombosis is witnessed essentially in the form of renal failure, coma, liver failure, respiratory failure, skin necrosis, gangrene and venous thromboembolism.[2] In chronic DIC, there may be very minimal or no clinical features or there may be only laboratory evidence of DIC.[7] Thus, we see marked heterogenicity in clinical manifestations, and the etiologic factor is the major predictor of the clinical events.[1]

In our case, perhaps an unspecified trivial trauma has instigated osteomyelitis of the tibia which remained undiagnosed till there was sepsis. A small post-traumatic right renal hematoma has gradually enlarged due to chronic DIC.

Though the pathophysiology is identical to acute DIC, clinical picture and laboratory findings in chronic DIC may be wavering. Here, nearly all of the global tests like platelet count, fibrinogen, PT and PTT may be normal. FDP, fibrinopeptide A and D dimer are usually raised and diagnostic.[7] Chronic DIC is usually associated with carcinomatosis, retained dead fetus, liver disease, aneurysm or hemangioma.[2] Sepsis usually causes acute DIC but nonovert chronic DIC is also observed.[1] Liver hematoma, subdural hematoma[3,4] and post-biopsy renal hematoma due to chronic DIC have been reported.[5]

Critical point in the management of DIC is to eradicate the primary disease and treat the concomitant causes.[8] Patients usually require treatment for thrombotic obstruction of the vasculature and subsequent multiorgan failure.[8] Theoretically, interruption of coagulation should be of benefit in patients with DIC. Heparin has been shown to have a beneficial effect in small, uncontrolled studies of patients with DIC, but not in controlled clinical trials.[9] The cornerstone of management of DIC is the treatment of the underlying disorder. Most often, these cases can be managed with blood and coagulation factors if the underlying cause is treated as in our case.
